# L-Threonine Supplementation During Colitis Onset Delays Disease Recovery

**DOI:** 10.3389/fphys.2018.01247

**Published:** 2018-09-05

**Authors:** Joana Gaifem, Luís G. Gonçalves, Ricardo J. Dinis-Oliveira, Cristina Cunha, Agostinho Carvalho, Egídio Torrado, Fernando Rodrigues, Margarida Saraiva, António G. Castro, Ricardo Silvestre

**Affiliations:** ^1^Life and Health Sciences Research Institute, School of Health Sciences, University of Minho, Braga, Portugal; ^2^ICVS/3B’s – PT Government Associate Laboratory, Guimarães, Portugal; ^3^Instituto de Tecnologia Química e Biológica António Xavier, Universidade NOVA de Lisboa, Oeiras, Portugal; ^4^IINFACTS – Institute of Research and Advanced Training in Health Sciences and Technologies, Department of Sciences, CESPU, CRL, University Institute of Health Sciences, Gandra, Portugal; ^5^UCIBIO, REQUIMTE, Laboratory of Toxicology, Department of Biological Sciences, Faculty of Pharmacy, University of Porto, Porto, Portugal; ^6^Department of Public Health and Forensic Sciences, and Medical Education, Faculty of Medicine, University of Porto, Porto, Portugal; ^7^Instituto de Investigação e Inovação em Saúde, Universidade do Porto, Porto, Portugal; ^8^Instituto de Biologia Molecular e Celular, Universidade do Porto, Porto, Portugal

**Keywords:** IBD, threonine, DSS-induced colitis, goblet cells, metabolomics, IL-22, mucin

## Abstract

Dietary nutrients have emerged as potential therapeutic adjuncts for inflammatory bowel disease (IBD) given their impact on intestinal homeostasis through the modulation of immune response, gut microbiota composition and epithelial barrier stability. Several nutrients have already been associated with a protective phenotype. Yet, there is a lack of knowledge toward the most promising ones as well as the most adequate phase of action. To unveil the most prominent therapy candidates we characterized the colon metabolic profile during colitis development. We have observed a twofold decrease in threonine levels in mice subjected to DSS-induced colitis. We then assessed the effect of threonine supplementation in the beginning of the inflammatory process (DSS + Thr) or when inflammation is already established (DSS + Thr D8). Colitis progression was similar between the treated groups and control colitic mice, yet threonine had a surprisingly detrimental effect when administered in the beginning of the disease, with mice displaying a delayed recovery when compared to control mice and mice supplemented with threonine after day 8. Although no major changes were found in their metabolic profile, DSS + Thr mice displayed altered expression in mucin-encoding genes, as well as in goblet cell counts, unveiling an impaired ability to produce mucus. Moreover, IL-22 secretion was decreased in DSS + Thr mice when compared to DSS + Thr D8 mice. Overall, these results suggest that supplementation with threonine during colitis induction impact goblet cell number and delays the recovery period. This reinforces the importance of a deeper understanding regarding threonine supplementation in IBD.

## Introduction

Inflammatory bowel disease (IBD) is a complex debilitating disorder of the gastrointestinal tract which comprises both Crohn’s disease and ulcerative colitis. Despite the unclear etiology of IBD, several factors have been accounted as key for the development of the disease, such as genetics, immune system and environmental factors, namely diet and gut microbiota composition (Khor et al., 2011).

Dietary supplementation has emerged as a promising therapeutic practice in the prevention and treatment of IBD (Durchschein et al., 2016). Recent evidence has revealed that fiber-enriched diets promote protection against IBD development, since dietary fiber is mainly fermented by intestinal microbiota into short-chain fatty acids (SCFAs), such as butyrate, acetate and propionate (den Besten et al., 2013). The protective properties of these metabolites are widely described by their impact on immune cell activation and epithelial barrier stability (Kelly et al., 2015; Macia et al., 2015), with decreased levels of SCFAs being found in colon samples from IBD patients (Huda-Faujan et al., 2010). Other studies have also pointed out several specific amino acids that can improve intestinal homeostasis, mainly by boosting mucosal healing and regeneration. For instance, glutamine is known to promote protection in dextran sulfate sodium (DSS)- and 2,4,6-trinitrobenzenesulfonic acid (TBNS)-induced intestinal inflammation, acting via NF-κB downregulation (Kretzmann et al., 2008). Colitic mice orally administered with glutamine displayed suppressive Th1/Th17 immune responses and subsequently decreased inflammation when compared to mice fed with regular diet (Hsiung et al., 2014). Other amino acids have been also associated with a protective phenotype against colitis. Using distinct animal models of colitis, diets enriched in threonine, serine, proline and cysteine, given before and throughout disease development, have been shown to restore mucin synthesis and stabilization of gut microbiota (Faure et al., 2006). Similar findings were observed with the administration of a mixture of threonine, methionine and monosodium glutamate after colitis induction (Liu et al., 2013).

Several studies have so far addressed whether administration of specific nutrients may arise as a prophylactic and/or therapeutic approach. However, there is a lack of knowledge toward the most promising and adequate phase of action. Thus, we investigated the metabolic profile of mice developing colitis, aiming to identify variation of metabolites during inflammation. The identification of the most attractive potential targets for therapy and the definition of a time range more prone to potentiate the effects of their supplementation may be relevant for future applications in IBD prophylaxis and therapy.

## Materials and Methods

### Animals

Seven to nine-week old C57BL/6J male mice were purchased from Charles River Laboratories and housed in i3S animal facilities, under pathogen free conditions, with food and water *ad libitum*. All experimental procedures were approved by the i3S Animal Ethics Committee and licensed by the Portuguese National Authority for Animal Health (DGAV) with reference 014811/2016-07-13.

### Colitis Induction

Dextran sulfate sodium (DSS; TdB Consultancy; 2% (w/v), molecular weight approximately 40000 Da) was administered in drinking water *ad libitum* for 5 days. Clinical signs of colitis were monitored daily and scored as a disease activity index (DAI; **Supplementary Table S1**).

### L-Threonine Administration

Mice were divided into DSS (control), DSS with L-Threonine (DSS + Thr) and DSS followed by L-Threonine administration at day 8 (DSS + Thr D8), as shown in **Figure 2A**. L-Threonine [Thr; Sigma–Aldrich; 0.166% (w/v) corresponding to 250 mg/Kg/day] was given in the drinking water *ad libitum*. The dose was chosen according to the daily intake in previous studies with rodents (Faure et al., 2006; Liu et al., 2013) and to a high-threonine human supplementation study (Pencharz et al., 2008). The safety of our protocol measuring biomarkers of renal and liver damage was evaluated to confirm the absence of toxicity (**Supplementary Figure S1**). Similar fluid intake was found among all groups.

### Metabolomic Analysis by Nuclear Magnetic Resonance (NMR)

Methanol/water extracts of colon were analyzed at an UltrashiedTM 800 Plus (Bruker) spectrometer as described in Graça et al. (2017). Metabolite concentrations were performed by integration of 1H-NMR resonances using TSP as reference.

### Quantitative Real-Time PCR (qPCR)

Total RNA was isolated from colonic samples (TripleXtractor, Grisp). As DSS inhibits both polymerase and reverse transcriptase activities, RNA was purified with lithium chloride, as in Viennois et al. (2013). qPCR was performed as described in Correia et al. (2017). The list of primers used is in **Supplementary Table S2**.

### Histology and Goblet Cell Count

Colons were fixed in 10% buffered formalin (Sigma–Aldrich) and embedded in paraffin. Sections of 5 μm were stained with hematoxylin/eosin and Alcian Blue/Periodic acid-Schiff. Goblet cell number was assessed for each experimental condition in a blinded fashion. Only crypts cut longitudinally from crypt opening to bottom were analyzed.

### Cytokine Quantification

Colonic explant cultures were performed as previously described (McNamee et al., 2011). Cytokine quantification was performed in supernatants by ELISA (Biolegend). Tissue explants were homogenized and total protein was measured using Bradford assay. The concentration of secreted cytokines in the supernatant was normalized to total tissue protein and expressed as picogram of cytokine *per* μg of total tissue protein.

### Statistical Analysis

Metabolite differences were evaluated by ANOVA in R statistical software. Partial least squares – discriminant analysis (PLS-DA) models were performed in SIMCA software. Other statistical analyses were carried out with GraphPad Prism (version 6.01). For multiple group comparisons one-way ANOVA test with a Tukey multiple-comparison posttest was performed, while for multiple group comparisons with repeated measures two-way ANOVA test with a Tukey multiple-comparison posttest was applied. Data are presented as mean ± standard deviation (SD). Statistically significant values are: ^∗^*p* < 0.05; ^∗∗^*p* < 0.01; ^∗∗∗^*p* < 0.001.

## Results and Discussion

**FIGURE 1:**
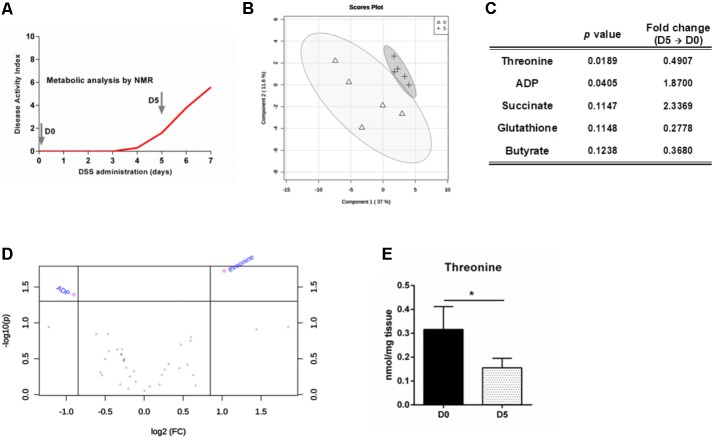
Threonine levels in the colon decrease with intestinal inflammation. **(A)** Mice were treated with DSS to induce colitis and colon metabolites were measured at day 0 and day 5 by NMR. **(B)** Partial least squares – discriminant analysis (PLS-DA) was obtained for samples of both days. **(C)** Top-five features identified by the combination of the *p*-value from *t*-test analysis and magnitude of the change (fold change from day 5 *versus* day 0). **(D)** Important metabolites selected by volcano plot with fold change threshold (x) 1.8 and *t*-test threshold (y) of 0.05. The purple circles represent features above the threshold. Both fold changes and *p-*values are log transformed. **(E)** Threonine levels in the colon at day 0 and day 5. Data is shown as mean ± SD; *n* = 5 mice/group. One representative experiment is shown out of two. ^∗^*p* < 0.05.

To characterize the colon metabolic profile during colitis development, metabolites present on colonic extracts of mice prior (day 0) and after 5 days of DSS-induced colitis were analyzed by NMR (**Figure 1A**). These time points were selected since it allows the comparison of a homeostatic profile (day 0) against a period with established inflammation and lesion, yet reversible and treatable (day 5). Multivariate analyses of the metabolomic data only demonstrated minor alterations between profiles for day 0 and day 5 of colitis development (**Figure 1B**). However, univariate analyses performed on the metabolites allow to discriminate ADP and particularly threonine as significantly altered from day 0 to day 5 (**Figures 1C,D**). Among essential amino acids, threonine has a prominent role in maintaining a healthy gut. Threonine is able to generate the main three SCFAs, namely acetate, butyrate and propionate (Neis et al., 2015). In fact, it has been previously identified several biosynthetic genes for threonine metabolism in the human gut microbiota, suggesting the relevance of this amino acid for microbiota biology (Abubucker et al., 2012). SCFAs are described as important modulators of immune response, since they are ligands for G-protein-coupled receptor 43 (GPR43) that is expressed by immune cells on the lamina propria, such as regulatory T cells, regulating the proinflammatory responses in the intestine (Bollrath and Powrie, 2013). Moreover, threonine is vastly metabolized in the intestine for mucin synthesis (Faure et al., 2005). These proteins are paramount in intestinal stability, since the mucus layer in the colonic outer layer prevents the direct contact of luminal microorganisms with the epithelium (Johansson et al., 2014). It has also been suggested that threonine requirements are increased under pathological settings to maintain proper intestinal function, such as production and formation of the mucus layer (Remond et al., 2009). Therefore, by participating in the mucus layer synthesis and production of anti-inflammatory SCFAs, threonine metabolism by gut microbiota proves to be essential for gut barrier integrity and function. Our data show that threonine levels drop 2-fold during colitis development until day 5 (**Figures 1C–E**). Therefore, we hypothesized that threonine supplementation during active inflammatory disease could help to restore the intestinal homeostasis and thus present some therapeutic potential.

**FIGURE 2:**
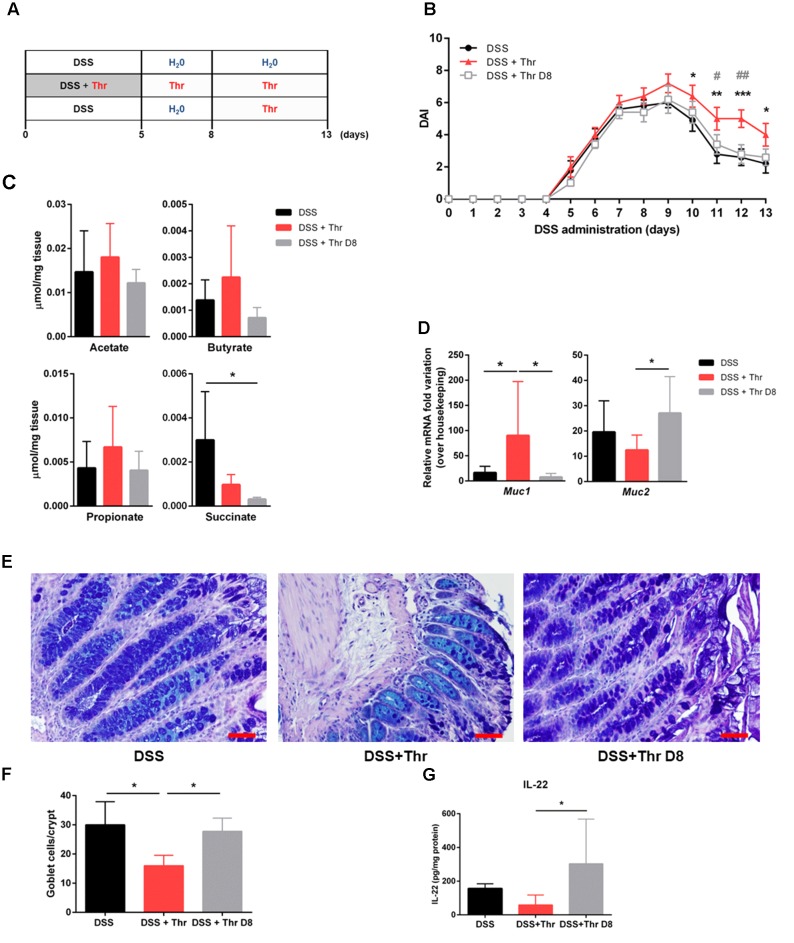
Threonine supplementation on the onset of colitis development delays recovery and decreases goblet cell number. **(A)** Mice were supplemented with threonine in the beginning of colitis induction (DSS + Thr) or after established inflammation (DSS + Thr D8). Mice treated only for colitis induction were used as control (DSS). **(B)** Disease progression was assessed by scoring the disease activity index (DAI) throughout the experiment. **(C)** Quantification of short-chain fatty acids (acetate, butyrate, propionate) and succinate by NMR was performed at day 13 of experiment. **(D)** Expression of mucin-encoding genes *Muc1* and *Muc2* was performed by qPCR at day 13. **(E)** Colonic tissue sections were stained with Alcian Blue/periodic acid-Schiff. **(F)** Quantification of goblet cell number per crypt. Scale bar = 50 μm. **(G)** IL-22 quantification on colonic explant cultures by ELISA. Data is shown as mean ± SD. *n* = 5 mice/group. One representative experiment is shown out of two. The symbol ^∗^ corresponds to statistical differences between DSS + Thr and DSS; while # is related to statistical differences between DSS + Thr and DSS + Thr D8. ^∗^*p* < 0.05; ^∗∗^*p* < 0.01; ^∗∗∗^*p* < 0.001.

Previous studies have investigated threonine in combination with other amino acids as a potential candidate for therapy against colitis (Faure et al., 2006; Liu et al., 2013). Nevertheless, not only single threonine supplementation was not evaluated before, but also there is scarce evidence regarding the most adequate time frame for its supplementation in colitis treatment. Accordingly, we addressed threonine supplementation in two distinct phases: (1) in parallel with the initiation of the inflammatory process, i.e., simultaneously with DSS administration (DSS + Thr), and (2) on day 8, when inflammation is already established (DSS + Thr D8). Mice subjected to DSS-induced colitis but without threonine supply were used as control (DSS) (**Figure 2A**). We observed that the three groups developed colitis with a similar progression profile. Nevertheless, after this time point, the recovery profile of colitic mice supplemented with threonine in the drinking water (DSS + Thr) was slower than that of the other two groups, showing statistically significant differences from day 10 to the final day of experiment when compared to control group (DSS). Besides, DSS + Thr mice had also a distinctive DAI score at day 11 and 12 when compared with mice that only received threonine after day 8 (DSS + Thr D8) (**Figure 2B**). Despite the divergent phenotype, no major differences were found in colon length neither in the intestinal permeability (**Supplementary Figure S1**).

Threonine administration was surprisingly detrimental for disease recovery when given at the setting of the inflammatory process. To understand this phenotype, we first evaluated the potential alterations in the colon metabolic profile among the different groups. No significant differences were found between most of the metabolites. Particularly, the levels of the major SCFAs (i.e., acetate, butyrate, and propionate), normally associated with a protective phenotype, were similar among groups. Only succinate levels, which is as an important marker of inflammation promoting IL-1β induction in inflammatory contexts (Tannahill et al., 2013), were found to be markedly decreased in DSS + Thr D8 mice (**Figure 2C**). No major alterations were observed in the inflammatory infiltrate profile, tissue organization and hepatic and renal toxicity serum biomarkers (**Supplementary Figure S1**).

Threonine plays a major role in mucin synthesis and consequently in the formation of the mucus layer. Indeed, lack of threonine is also known to impair intestinal paracellular permeability and is associated with fewer goblet cells and mucus synthesis (Faure et al., 2005; Mao et al., 2011). The mucus layer serves as a barrier against microbial translocation to the lamina propria and therefore its integrity is paramount for intestinal homeostasis. When we analyzed the expression of mucin-encoding genes, we found that *Muc2* expression is decreased in DSS + Thr mice when compared to mice that only received threonine at day 8 (DSS + Thr D8) (**Figure 2D**). *Muc2* encodes for the oligomeric mucus gel-forming mucin 2 protein that is the major responsible for mucus synthesis. Indeed, impairment or total absence of the mucus layer is associated with severe colitis, as observed in *Muc2*-deficient mice (Van der Sluis et al., 2006). We also found that DSS + Thr mice display higher expression of *Muc1*, which has shown to contribute to intestinal inflammation and colon cancer progression (Baldus et al., 2004; Takahashi et al., 2015). We next quantified the number of goblet cells of the colonic mucosa. These are a secretory epithelial cell lineage found in both the small and the large intestines, whose major function is the production of mucus. By analyzing colon slices stained with Alcian Blue/Periodic acid-Schiff, we observed that DSS + Thr mice displayed significantly fewer goblet cells when compared to both DSS and DSS + Thr D8 (**Figures 2E,F**). Therefore, our results suggest that an alteration in mucus synthesis due to threonine administration during the onset of disease may impact intestinal integrity, by delaying the recovery of disease. Faure et al. (2006) have demonstrated that supplementation with diet containing higher doses of amino acids, including L-threonine, lead to an increase in goblet cell number, regulated mucin production in the colon and restored microbiota composition after DSS treatment in rats. Notwithstanding, not only the animal model is different, but also L-threonine was given before colitis induction, which may be underlying the distinctive outcome.

Previous studies have linked several cytokines to mucus production in the intestine (Parks et al., 2015). To examine the immunological profile of the three groups, cytokine levels in the colon were quantified. No major changes were observed between the groups for interleukin (IL)-1β, IL-12p70, IL-10, IL-17A/F and granulocyte-macrophage colony-stimulating factor (GM-CSF) levels (**Supplementary Figure S2**). Notwithstanding, the amount of IL-22 was significantly decreased in DSS + Thr mice when compared to DSS + Thr D8 mice (**Figure 2G**). IL-22 is a member of the IL-10 family of cytokines and has been vastly studied in the context of intestinal homeostasis. It can be produced by several cell types, such as T helper (Th1) 1, Th17, Th22 and innate lymphoid cells (ILCs), and present several roles in the gastrointestinal tract, such as tissue regeneration and maintenance of the intestinal epithelial barrier (Rutz et al., 2013). Thus, the decreased IL-22 levels may be associated with delayed recovery of the intestinal balance.

Overall, our data demonstrate that supplementation of threonine during colitis induction impairs goblet cell number, with concomitant decreased *Muc2* expression and IL-22 production. These variations are likely to be the cause of delayed recovery observed in this situation. Interestingly, these effects are not seen when threonine is administered once colitis is established. Acute DSS-induced colitis is known to promote gut microbial dysbiosis (Munyaka et al., 2016). Threonine is metabolized by some intestinal commensal bacteria, leading to the production of several metabolites used for intestinal maintenance and to mediate immune responses (Neis et al., 2015). Thus, threonine supplementation during induction of colitis may impact differently the colonic microbiota populations present during the onset and upon the establishment of inflammation, having ultimately distinct effects in intestinal function. Further understanding of the mechanisms underlying threonine supplementation may give new insights on how dietary nutrients modulate the dynamic balance between microbiome, immune response and barrier function.

## Ethics Statement

This study was carried out in accordance with the recommendations of European Council Directive (2010/63/EU) guidelines that where transposed into Portuguese law (Decree-Law n.°113/2013, August 7th), i3S Animal Ethics Committee and licensed by the Portuguese National Authority for Animal Health (DGAV). The protocol was approved by the i3S Animal Ethics Committee and licensed by the Portuguese National Authority for Animal Health (DGAV) with reference 014811/2016-07-13.

## Author Contributions

JG, ET, CC, AgC, FR, MS, AnC, and RS designed the experiments. JG, LG, RD-O, and RS performed the experiments. JG, LG, RD-O, and RS analyzed the data. JG, LG, RD-O, and RS interpreted the results. JG and RS drafted the manuscript and prepared the tables and figures. JG, LG, RD-O, ET, CC, AgC, FR, MS, AnC, and RS revised the paper and approved the final version of the manuscript.

## Conflict of Interest Statement

The authors declare that the research was conducted in the absence of any commercial or financial relationships that could be construed as a potential conflict of interest. The reviewer DS and handling Editor declared their shared affiliation at the time of the review.
