# Supplementary material for: L-Threonine Supplementation During Colitis Onset Delays Disease Recovery
Source: Front Physiol. 2018 Sep 5;9:1247. doi: 10.3389/fphys.2018.01247 (PMC6134202; doi:10.3389/fphys.2018.01247)
Supplement: Supplementary file 1 [file Data_Sheet_1.docx]

**Supplementary methods**

***In vivo* intestinal permeability assay**

To assess barrier function at day 13, *in vivo* intestinal permeability assay was performed by FITC-labelled dextran administration. Food and water were withdrawn for eight hours and mice were administered with 44 mg/100 g body weight of FITC-labelled dextran (TdB Consultancy; 4 kDa) by oral gavage. Serum was collected four hours later and fluorescence intensity was measured by spectrophotofluorimetry (excitation: 485 nm; emission: 528 nm). Concentrations were determined using a standard curve of serially diluted FITC-labelled dextran. Serum from mice not administered with the permeability tracer was used as background control.

**Quantification of serum toxicological biomarkers**

Aspartate transaminase (AST), alanine aminotransferase (ALT) and creatinine were measured on an AutoAnalyzer (PRESTIGE 24i, PZ Cormay S.A.).

**Supplementary Tables and Figures**

**S1 Table** – Disease activity index (DAI) scores for colitis evaluation. The final score is obtained by the sum of each parameter.

| **Score** | **Weight loss** | **Stool consistency** | **Bleeding** |
| --- | --- | --- | --- |
| 0 | No loss | Normal | No blood |
| 1 | 1-5% | Mild-soft | Brown color |
| 2 | 6-10% | Very soft | Reddish color |
| 3 | 11-20% | Diarrhea | Bloody stool |
| 4 | > 20% |  | Gross bleeding |

**S2 Table** – List of primers used for qPCR.

| **Genes** | **Forward sequence** | **Reverse sequence** |
| --- | --- | --- |
| ***Muc1*** | CCCTATGAGGAGGTTTCGGC | AAGGGCATGAACAGCCTACC |
| ***Muc2*** | TCCTGACCAAGAGCGAACAC | ACAGCACGACAGTCTTCAGG |
| ***Ubq*** | TGGCTATTAATTATTCGGTCTGCAT | GCAAGTGGCTAGAGTGCAGAGTAA |


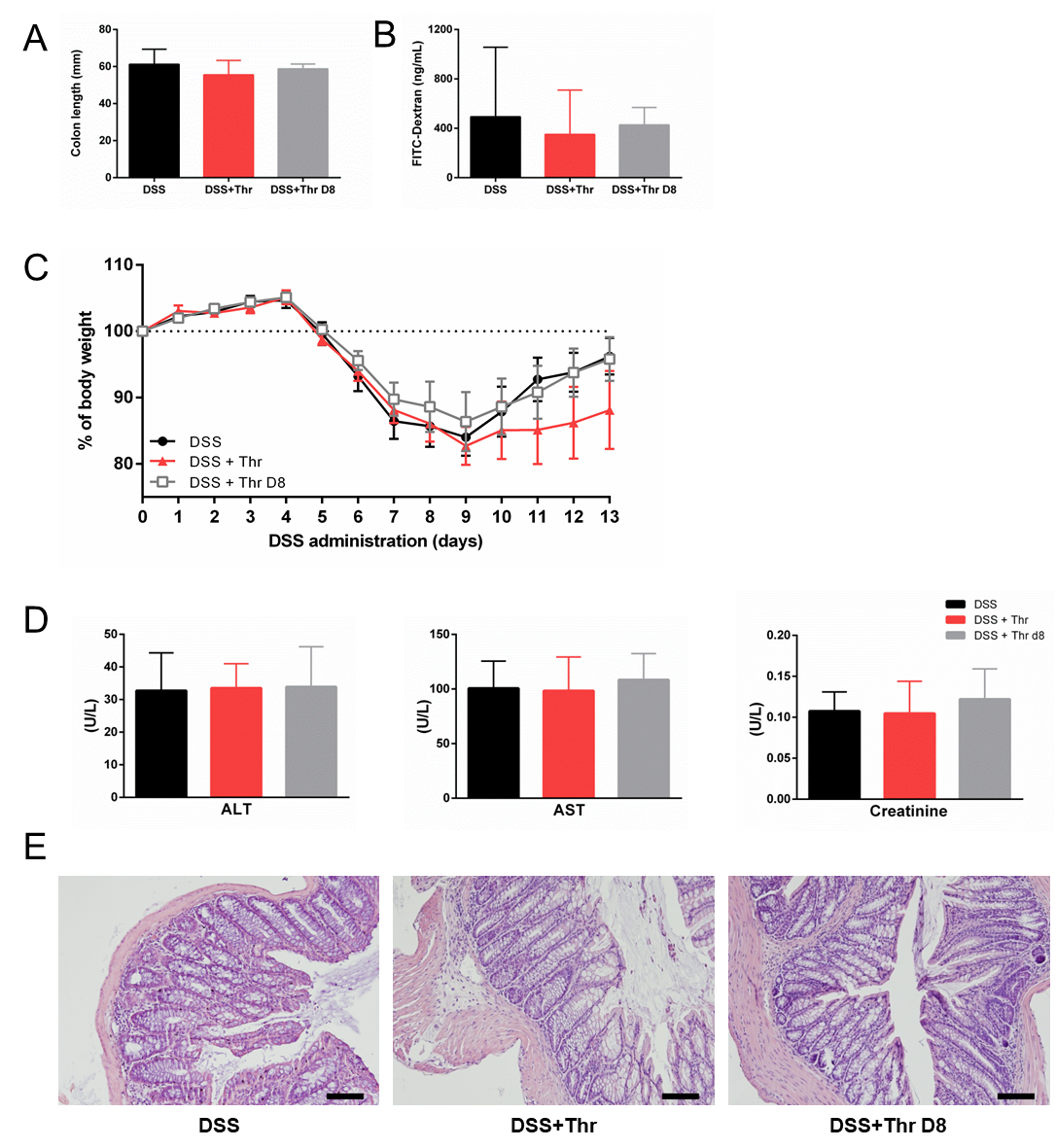


**Figure S1 - Evaluation of histological and clinical parameters.** (A) Colon length was measured after excision at day 13. (B) Intestinal permeability was measured after administration of FITC-Dextran by oral gavage and quantified in the serum after four hours of administration. (C) As one of the parameters of DAI, the body weight was measured daily during the experimental procedure. (D) Serum levels of alanine transaminase (ALT), aspartate aminotransferase (AST) and creatinine were analyzed for renal and hepatic toxicity control. (E) Histological analysis of hematoxylin & eosin staining of mice treated with DSS, DSS+Thr and DSS+Thr D8. Scale bar = 100 µm. Data is shown as mean ± SD; n=5 mice/group. No statistical differences were found between conditions.


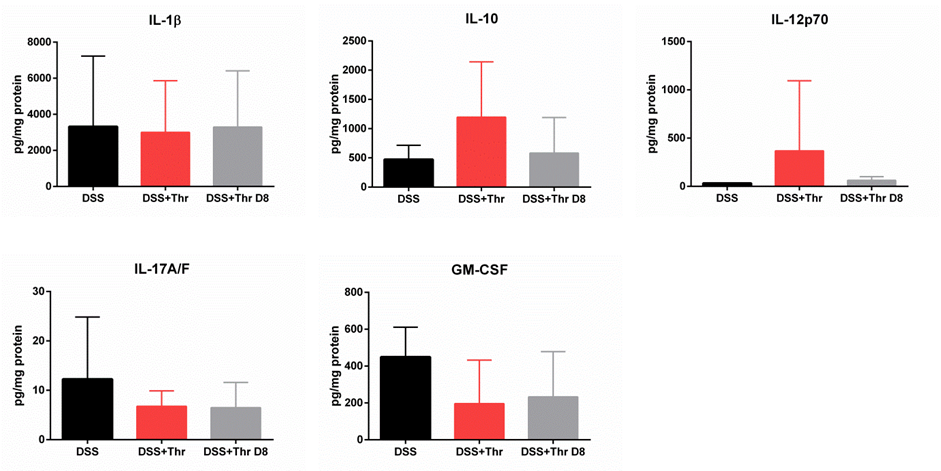


**Figure S2 - Quantification of cytokine levels in supernatants from colonic extracts.** Levels of colonic IL-1β, IL-10, IL-12p70, IL-17A/F and GM-CSF were measured in the supernatants of ex vivo colonic explantes cultured at day 13. The concentration of secreted cytokines in the supernatant was normalized to total explant protein and expressed as picogram of cytokine per milligram of protein. Data is shown as mean ± SD; n=5 mice/group. No statistical differences were found between conditions.
